# Oxidative stress markers in hypertensive states of pregnancy: preterm and term disease

**DOI:** 10.3389/fphys.2014.00310

**Published:** 2014-08-25

**Authors:** Lesia O. Kurlak, Amanda Green, Pamela Loughna, Fiona Broughton Pipkin

**Affiliations:** Department of Obstetrics and Gynaecology, School of Medicine, University of Nottingham, City HospitalNottingham, UK

**Keywords:** hypertension, pregnancy, pre-eclampsia, oxidative stress, term, preterm

## Abstract

Discussion continues as to whether *de novo* hypertension in pregnancy with significant proteinuria (pre-eclampsia; PE) and non-proteinuric new hypertension (gestational hypertension; GH) are parts of the same disease spectrum or represent different conditions. Non-pregnant hypertension, pregnancy and PE are all associated with oxidative stress. We have established a 6 weeks postpartum clinic for women who experienced a hypertensive pregnancy. We hypothesized that PE and GH could be distinguished by markers of oxidative stress; thiobarbituric acid reactive substances (TBARS) and antioxidants (ferric ion reducing ability of plasma; FRAP). Since the severity of PE and GH is greater pre-term, we also compared pre-term and term disease. Fifty-eight women had term PE, 23 pre-term PE, 60 had term GH and 6 pre-term GH, 11 pre-existing (essential) hypertension (EH) without PE. Limited data were available from normotensive pregnancies (*n* = 7) and non-pregnant controls (*n* = 14). There were no differences in postpartum TBARS or FRAP between hypertensive states; TBARS (*P* = 0.001) and FRAP (*P* = 0.009) were lower in plasma of non-pregnant controls compared to recently-pregnant women. Interestingly FRAP was higher in preterm than term GH (*P* = 0.013). In PE and GH, TBARS correlated with low density lipoprotein (LDL)-cholesterol (*P* = 0.036); this association strengthened with inclusion of EH (*P* = 0.011). The 10 year Framingham index for cardiovascular risk was positively associated with TBARS (*P* = 0.003). Oxidative stress profiles do not differ between hypertensive states but appear to distinguish between recently-pregnant and non-pregnant states. This suggests that pregnancy may alter vascular integrity with changes remaining 6 weeks postpartum. LDL-cholesterol is a known determinant of oxidative stress in cardiovascular disease and we have shown this association to be present in hypertensive pregnancy further emphasizing that such a pregnancy may be revealing a pre-existing cardiovascular risk.

## Introduction

Reactive oxygen species (ROS) are molecules produced from the reduction of molecular oxygen, generated as by-products of aerobic respiration and metabolism. These molecules have diverse chemical properties and are capable of activating various different signaling pathways. Major effects of ROS involve the regulation of physiological processes including cell growth and differentiation, modulation of extracellular matrix production and breakdown, as well as inactivation of nitric oxide and stimulation of kinases (Paravicini and Touyz, 2006). ROS are important in these processes but if unchecked, can have destructive effects on cell components; damaging membranes by peroxidation of long chain fatty acids, oxidation and nitration of proteins, and the occurrence of DNA lesions/strand breaks. To counterbalance the ROS, cells have endogenous antioxidant systems in the form of vitamins C and E, enzymes such as the superoxide dismutase (SOD), catalase and glutathione peroxidase (GPxs) families, polyphenols and trace elements including copper, zinc, manganese and selenium (Mistry and Williams, 2011).

Oxidative stress occurs when the production of ROS overwhelms the antioxidant capacity resulting in overall damage to cells and has been implicated in the pathology of many hypertensive pregnancy-related conditions including pre-eclampsia (PE) and gestational hypertension (GH) (Burton and Jauniaux, 2011). Placentation occurs at low oxygen tensions up to 10 weeks of gestation (Rodesch et al., 1992; Jauniaux et al., 2000), a hypoxic environment being essential for trophoblast proliferation and invasion of the maternal spiral arteries for the restructuring of these blood vessels. The successful completion of spiral artery remodeling at around 10–12 weeks' gestation, results in unplugging of these vessels, which have been transformed into flaccid conduits with no resistance, enabling uteroplacental blood to flow unimpeded (Pijnenborg et al., 1991, 2006). This restoration of blood flow results in a rapid rise in the tissue oxygen tension which triggers the production of human chorionic gonadotrophin (hCG) and enzymes such as P-450 cytochrome aromatase, involved in the synthesis of oestrogens (Jauniaux et al., 2006); this gives rise to acceleration of placental tissue growth. The rapid increase in oxygenation will also stimulate production of ROS and concomitant increases in the production and activity of antioxidant enzymes (Jauniaux et al., 2006).

However, in pathological conditions such as PE, one of the underlying mechanisms of this condition is the shallow invasion of trophoblasts during these early stages of placentation. Incomplete remodeling of the spiral arteries leads to intermittent, more pulsatile, blood flow giving rise to ischaemia/reperfusion–type injury (Hung and Burton, 2006) with subsequent increases in ROS. Antioxidant capacity (Madazli et al., 2002; Mistry et al., 2008, 2010) is lower than in normal pregnancy, further augmenting the ROS concentration with the subsequent rise in oxidative stress so characteristic of PE and other hypertensive-pregnancy disorders. This shift in antioxidant capacity is a consequence of inadequate micronutrient status of zinc, manganese and selenium (Mistry and Williams, 2011) which contribute to the enzyme activity of antioxidant superoxide dismutases and glutathione peroxidases (Mistry et al., 2008).

There is an ongoing debate as to whether *de novo* hypertension in pregnancy associated with raised protein excretion (PE) is a different condition to non-proteinuric new hypertension (GH) or whether it is indeed a different part of a spectrum of the same disease. GH is defined as elevated blood pressure (SBP ≥ 140 mm Hg or DBP ≥ 90 mm Hg) after 20 weeks of gestation in a previously normotensive woman (Task Force on Hypertension in Pregnancy, 2013) without proteinuria whereas PE is defined as *de novo* hypertension with significant proteinuria where there is no evidence of a urinary tract infection (Brown et al., 2001). Women who experience GH at term are phenotypically similar to those with mild essential hypertension (EH) and pregnancy could be acting as a stress test and unmasking this condition (Williams, 2003). The risk factors for PE and GH are similar (e.g., type I and gestational diabetes, high body mass index, twin birth), implying similarities in the biological mechanisms underlying the development of these conditions, i.e., endothelial dysfunction and inadequate placental perfusion (Ros et al., 1998). Moreover, between 20 and 50% of women with early presentation of GH (<36 weeks) go on to develop PE (Saudan et al., 1998).

There is increasing evidence to suggest that not only are PE and GH detrimental to the pregnant woman, posing an increased risk of the accelerated development of cardiovascular disease (CVD; odds ratio 2.28), cerebrovascular injury (odds ratio 1.76), (Brown et al., 2013) or end stage renal disease (OR 10.64) (Wu et al., 2014) they are also associated with sequelae for her offspring, with a doubling of risk of stroke (odds ratio 1.90) in adult life (Kajantie et al., 2009; Sutherland et al., 2014). Although this risk to the mother is not further compounded by a preterm delivery, early parturition clearly does have consequences for the development of the infant. Low birthweight is known to be associated with increased rates of coronary heart disease and the related disorders, stroke, hypertension and type 2 diabetes (Barker, 2006). Therefore, there is a serious need to follow up women who have experienced hypertensive pregnancies even if the pregnancy continues to term.

As part of a larger study following up women with hypertensive pregnancies (GH, PE, and EH without superimposed PE), the aims of this present study were to address the specific questions of whether or not markers of oxidative stress are still raised in maternal blood at 6 weeks postpartum and whether their concentrations differ between the hypertensive states. We hypothesized that oxidative stress markers would still be higher following the more severe hypertensive states, since this would reflect the mother's basic diet and her capacity to synthesize antioxidants. We assumed that there would no major change in diets before and after delivery; women with the severest hypertension would already have inadequate dietary intake and/or inadequate capacity to synthesize endogenous antioxidants such as the glutathione peroxidases and thus already have an imbalance between ROS production and antioxidant defenses. In addition, since severity of PE and GH is greater pre-term (Sibai, 2003), it was also postulated that oxidative stress profiles would differ in pre-term and term disease with higher residual oxidative stress in the women who had delivered preterm.

## Methods

### Subjects

All participants provided informed written consent under ethical approval granted by the Nottingham University Hospitals Research and Development department (ref 10OB002).

Eligible women were recruited to the study at the City Hospital Maternity Unit between January 2011 and February 2012. All women attending the unit, who were identified as having hypertension during their pregnancy, were offered a 6-week appointment at the postnatal hypertension research clinic.

Hypertension was defined as blood pressure (BP) of >140/90 mmHg on two or more occasions >4 h apart; significant proteinuria ≥0.3 g urinary protein per 24 h or a spot protein:creatinine ratio (PCR) of over 30 or ≥2+ protein on urine dipstick, with no evidence of a urinary tract infection. The diagnostic groups were classified according to the International Society for the Study of Hypertension in Pregnancy (ISSHP) guidelines as follows (Brown et al., 2001): preeclampsia (PE)—new hypertension and significant proteinuria ≥20 weeks gestation; gestational hypertension (GH)—new hypertension ≥20 weeks gestation in the absence of significant proteinuria; essential hypertension (EH)—hypertension ≤20 weeks gestation or in women already taking anti-hypertensive medication at booking.

The number of women included in each category were PE: *n* = 81(term *n* = 58, preterm *n* = 23); GH: *n* = 66 (term *n* = 60, preterm *n* = 6); EH without superimposed PE: *n* = 11. Limited control data on FRAP and TBARS, measured in our laboratory, were available, also at the 6 weeks postnatal visit, from 7 women who had enjoyed a normotensive pregnancy and from 14 non-pregnant control women.

### Laboratory investigations

During the visit, routine clinical laboratory measurements were carried out; for this sample set the following data are included: maternal blood pressure, LDL-cholesterol, HDL-cholesterol, total cholesterol. The women were only 6 weeks postpartum. It was felt that their compliance with a request to attend the clinic fasting would be very low. The various plasma cholesterol measurements are thus random, not fasting.

### Oxidative stress markers

#### Thiobarbituric acid-reactive substances (TBARS)

The measurement of TBARS is a well-established method for determination of lipid-peroxidation utilizing the reaction between malondialdehyde (MDA), a product of lipid peroxidation, and thiobarbituric acid (TBA) at temperatures of 90–100°C. The pink MDA-(TBA) 2 complex formed is quantified spectrophotometrically at 530–540 nm. Results are expressed in μmol of MDA equivalents. Plasma samples (100 μl) were assayed neat, without dilution and in triplicate using a commercial kit (Cayman Chemicals 10009055) according to a modified version of the manufacturer's protocol. Following the reaction at high temperature, the vials were centrifuged for 15–20 min, longer than the recommended time and at room temperature rather than 4°C: this minimized cloudiness in the samples which interfered with the optical density assessments. The intra- and inter-assay coefficients of variation were 2.1 and 9.1% respectively.

#### Ferric reducing ability of plasma (FRAP)

The ferric reducing ability of plasma assay was based on the method of Benzie & Strain (Benzie and Strain, 1996). At low pH a ferric-tripyridyltriazine (Fe^III^TPTZ) complex is reduced to the ferrous (Fe^II^) form provided a reductant (antioxidant) is present. In the FRAP assay, excess Fe^III^ is used so that the rate-limiting factor of the production of the Fe^II^ form of the complex is the reducing ability of the plasma sample. Ascorbic acid is used as a standard and has a known FRAP value of 2, the workable assay range was 62.5–1000 μM. A reagent mix consisting of tripyridyltriazine, ferric chloride and acetate buffer was added to 10 μl plasma samples, assayed at a dilution of 1:2, and incubated at 37°C. Absorbance of the solution was measured at 593 nm at time intervals up to 8 min and the change in absorbance calculated and compared to the standard.

All samples were run blinded as to group and in triplicate. In our laboratory, the intra- and inter-assay coefficients of variation were 6 and 13.4% respectively.

### 10 year Framingham index

This index has been developed from the study that originated in 1948 in Framingham, USA (Kannel et al., 1976). A risk score for developing CVD is calculated from a combination of age, gender, BP, cholesterol levels, diabetes and cigarette smoking. Historically, this has been the most commonly used risk score in the developed world.

### Statistical analyses

All tests were performed using IBM SPSS version 21. Data were tested for normality of distribution using the Kolmogorov-Smirnov test and appropriate distributional plots. Summary data are presented as mean [SD] or median [quartiles] as appropriate for the distribution. Between-group comparisons were made using Mann-Whitney *U*-tests. Spearman's Rank correlation test was used to test for associations between variables.

## Results

### Subjects

The basal characteristics of the participants are shown in Table 1.

**Table 1:** **Maternal demographic and laboratory parameters**.

	**PE (*n* = 81)**	**GH (*n* = 66)**	**EH, no PE (*n* = 11)**	**Normotensive (*n* = 7)**
Maternal age (years)	29.6 ± 5.9	30.02 ± 6.6	32.2 ± 4.1	29.1 ± 3.7
Gestation at delivery (weeks)	38.0 ± 2.6	39.3 ± 1.9	38.5 ± 1.9	39.7 ± 1.3
BMI (kg/m^2^)	28.8 ± 5.7	30.8 ± 6.2	30.6 ± 7.2	25.2 ± 6.02
Systolic BP (mmHg)	122.3 ± 11.3	127.8 ± 11.4	135.7 ± 16.7^**^	105.6 ± 11.9
Diastolic BP—K4 (mmHg)	81.7 ± 8.2	86.2 ± 9.7	92.5 ± 16.1^**^	68.6 ± 10.8
Smokers [n (%)]	(5) 6.2	(5) 7.6	(1) 9.0	(1) 14.0
Breast feeding (%)	32	28	43	71
Antihypertensive medication (%)	10.0	9.2	78.6	0
Total-cholesterol (mmol/L)	4.9 [4.6–5.5]	5.0 [4.5–5.5]	5.4 [5.15–5.8]	5.3 [5.25–5.6]
LDL-cholesterol (mmol/L)	1.5 [1.3–1.7]	1.5 [1.3–1.7]	1.8 [1.7–2.0]^*^	1.4 [1.4–2.05]
HDL-cholesterol (mmol/L)	2.9 [2.4–3.3]	2.8 [2.4–3.3]	3.0 [2.8–3.6]	3.4 [3.05–3.6]
Framingham 10 year CVD risk score	0.69 [0.47–1.08]	0.81 [0.53–1.38]	1.62 [1.28–1.77]^**^	0.44 [0.38–0.61]

*Values expressed as means ± SD or Median [interquartile range]*,

* P < 0.05;

** *P < 0.005*.

Term delivery was defined as >36 weeks. In the PE pregnancies there were 58 term deliveries, median gestational age 39 [IQR 38.3–40.3] and 23 preterm, median gestational age 34.9 [34.4–35.9]; GH term, *n* = 60, median gestational age 40.0 [38.9–40.9] and GH preterm, *n* = 6, median gestational age 35 [33.7–36.9].

The non-pregnant controls (*n* = 14) had a median age and BMI of 21 [20–23] years and 23.4 [21.7–26.2] kg/m^2^ respectively. All were nulliparous.

### Oxidative stress marker (TBARS)

**Figure 1:**
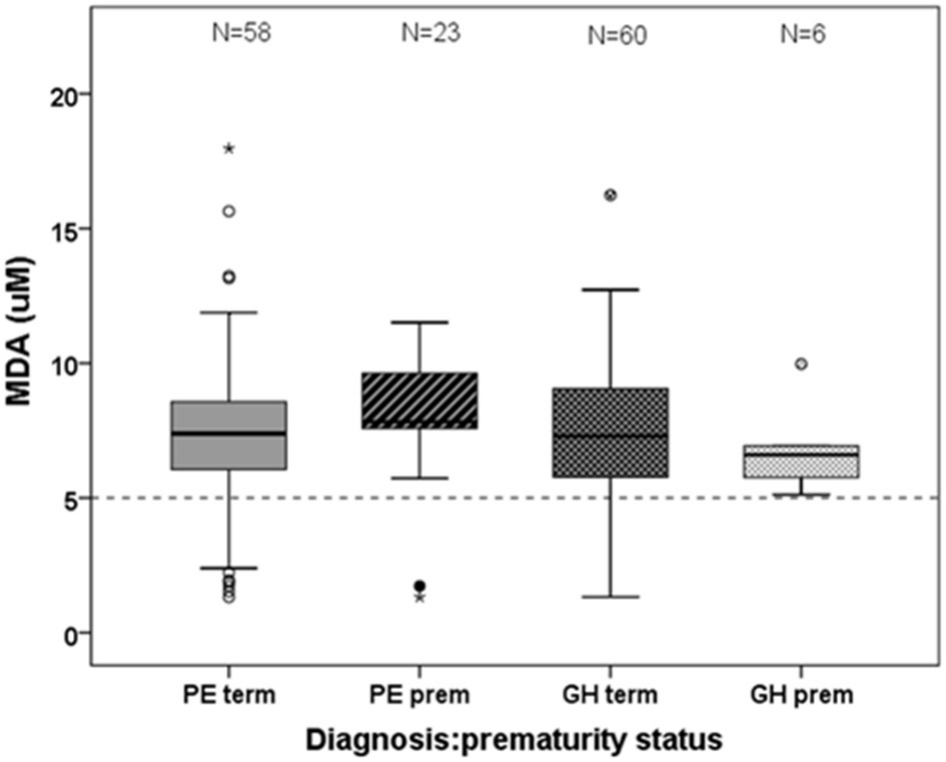
**Lipid peroxidation, measured in units of MDA, shown by prematurity status in the PE and GH diagnostic groups**. No differences were observed between hypertensive diagnostic groups or between term and preterm status. The dotted line is the median reference value for non-pregnant females within this assay.

At 6 weeks postpartum, there were no statistically-significant differences in the median maternal TBARS concentration between the hypertensive pregnancy groups: PE 7.6 [IQR 6.1–8.7]; GH 7.3 [5.8–8.6] and EH without PE, 10.5 [7.2–12.4] μM. Mothers who had delivered preterm (<36 weeks) had comparable plasma TBARS to mothers who had progressed to term (Figure 1). The median TBARS concentration in the normotensive pregnancy controls was very similar to that in the PE and GH groups (7.3 [7.1–9.1] μM). However, the median concentration was significantly lower (4.8 [4.2–6.1] μM) in the non-pregnant controls than in any of the recently-pregnant groups (*P* < 0.001).

### Antioxidant capacity (FRAP)

**Figure 2:**
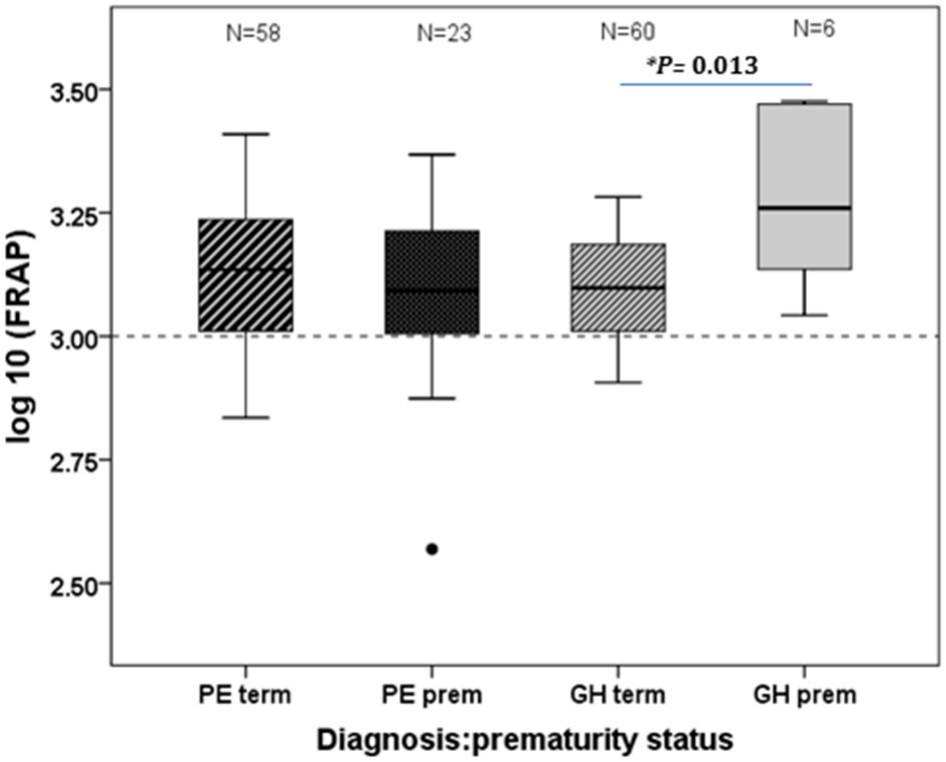
**Antioxidant status, measured as FRAP, shown by prematurity status in the PE and GH diagnostic groups**. Note the log_10_ scale. Significantly higher antioxidant status in the GH preterm than the GH term group (*P* = 0.013). The dotted line is the median reference value for non-pregnant females within this assay.

The median overall measure of plasma antioxidant capacity, FRAP (μM), was also similar in the various categories of hypertensive pregnancy: PE 1004.4 [IQR 836.6–1282.6]; GH 999.8 [874.0–1252.9] and EH without PE, 905.0 [790.4–1181.4]. Comparing term and preterm PE and GH, FRAP was significantly higher in the preterm-GH (*P* = 0.013), (Figure 2).

The normotensive pregnancy controls had significantly higher median FRAP (*P* = 0.001) 1209 [1182–1409] than non-pregnant controls, median 1002 [947–1043]. FRAP concentrations were significantly lower in the plasma of women who had never been pregnant compared to the recently-pregnant women (*P* = 0.009).

### Associations with cardiovascular risk

**Figure 3:**
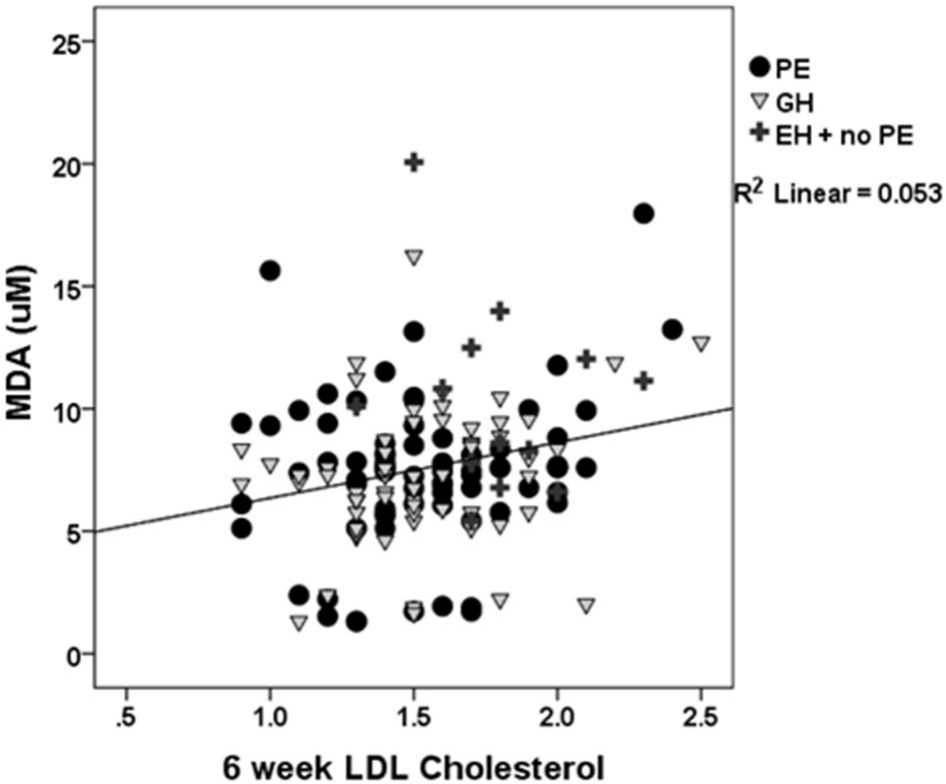
**The relationship between oxidative lipid peroxidation (TBARS-MDA) and LDL-cholesterol at 6 weeks postpartum**. A significant positive association was found (*R*^2^ = 0.053, *r* = 0.23, *P* = 0.011).

Possible associations with known cardiovascular risk factors were explored. In PE and GH (pregnancy-specific), there was a significant correlation between TBARS and LDL-cholesterol at the 6 week visit (*P* = 0.036; Figure 3): if EH (no PE) was also included, the correlation was even stronger (*r* = 0.22, *P* = 0.011). This relationship was not apparent when total cholesterol concentrations were considered.

**Figure 4:**
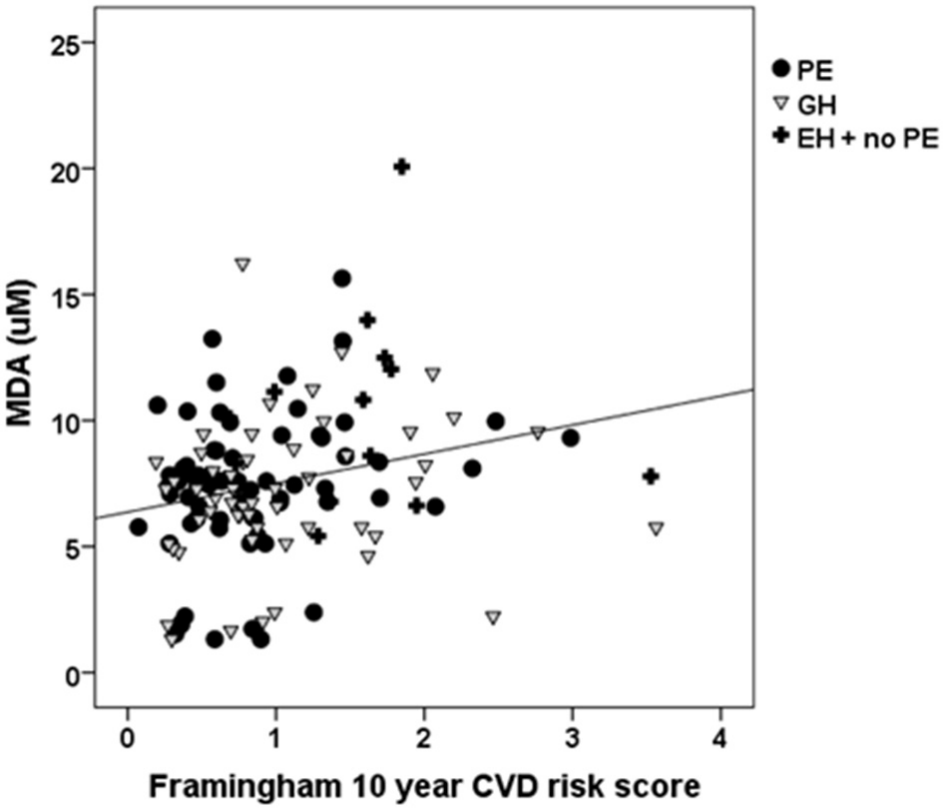
**The relationship between lipid peroxidation, measured by TBARS-MDA, at 6 weeks postpartum, and a cardiovascular disease risk score, the Framingham 10 year index (*R*^2^ = 0.061, *r* = 0.248, *P* = 0.003)**.

The 10 year Framingham Index is one of two most commonly used in the UK and is still currently included in the NICE guidelines on primary prevention of CVD. Although the participants enrolled on this study were younger than the Framingham cohort, it was felt to be the best available index to use. Overall the three hypertensive categories (PE, GH, and EH without PE), the 10 year Framingham Index showed a positive association with plasma TBARS (*r* = 0.248, *P* = 0.003; Figure 4). No such association was observed in the women who had a normotensive pregnancy.

## Discussion

This is one of the first large-scale investigations to follow up hypertensive pregnancies, postpartum. Our analysis of 158 women has demonstrated that TBARS, as markers of serum oxidative stress, are elevated 6 weeks after delivery compared to non-pregnant female reference values. This suggests that the impact of pregnancy on lipid peroxidation may have longer-lasting effects than previously thought. The lack of distinction between the different hypertensive disorders, whether studied overall or in relation to the timing of disease onset, was contrary to our hypothesis. However, although, antioxidant capacity, as measured by FRAP, also did not differ between hypertensive diagnostic groups overall, when assessed 6 weeks postpartum, FRAP was significantly higher in preterm than term GH (Figure 2). It was not possible to identify which component of the antioxidant spectrum was responsible for this increase, but we speculate that it might represent an increase in synthesis of endogenous antioxidants, as a protective response to the hypertension. Interestingly, median FRAP in all groups hypertensive in pregnancy remained somewhat lower than in the controls with normotensive pregnancy at 6 weeks postnatal.

Previous studies of oxidative stress markers during pregnancy or at the time of delivery have used comparable measures such as lipid hydroperoxide and malondialdehyde concentration (Morris et al., 1998), total antioxidant capacity or biological antioxidant potential (Hsieh et al., 2012; Gomes et al., 2013; Watanabe et al., 2013). There is a substantial body of evidence showing that PE is a state of elevated placental oxidative stress (Burton and Jauniaux, 2011). The rise in circulatory markers is believed to originate from this placental generation of ROS, contributing to the ROS generated during the systemic inflammatory response (Borzychowski et al., 2006). More specific markers of oxidative stress, such as plasma 8-isoprostane concentrations, are higher in hypertensive pregnancies compared to normal pregnancy, sampled at late gestation or delivery (Hubel, 1999; Rogers et al., 2006; Bazavilvaso-Rodriguez et al., 2011). As the pathology of endothelial dysfunction is a shared characteristic of the hypertensive disorders of pregnancy, it is not entirely surprising that a general measure of lipid peroxidation does not distinguish between the differing clinical states. Lipid peroxidation values begin to fall within 24 h of delivery (Little and Gladen, 1999; Gomes et al., 2013). However, the fact that lipid peroxidation appears still to be higher, at 6 week postpartum, in women who had essential hypertension, as compared to those who had normotensive pregnancies, suggests that a degree of underlying damage may have been present before pregnancy in these women. As the majority of women with EH continued to need antihypertensive medication at the time of the postnatal visit, we repeated our initial analysis and found that continuing medication was not associated with any change in either TBARS or FRAP. There are no known reports of these antihypertensive medications (labetalol/methyldopa/ atenolol) affecting markers of oxidative stress *per se* (Silva et al., 2011). Only one study reports the calcium channel blocker - nifedipine, as having a lowering effect on TBARS (Sankar et al., 2005) and then only if given in combination with sesame oil. It can be seen in Figures 3, 4 that a group of women, who had both PE and GH, had evidently lower plasma MDA concentrations than the remainder; we were unable to identify any other distinguishing feature in these women.

Pregnancy *per se* leads to an increased oxidative burden as high maternal and fetal oxygen demand increases oxygen metabolism (Morris et al., 1998; Roes et al., 2006). Longitudinal studies of oxidative stress and antioxidant status in pregnancy (Little and Gladen, 1999; Hung et al., 2010), indicate that lipid peroxides rise in pregnancy with highest levels of peroxidation markers being in the 2nd trimester and either being maintained or declining slightly in the 3rd trimester. Antioxidant status as measured by FRAP, has been shown to be lower than non-pregnant levels when considering gestational age earlier than 30 weeks, before increasing toward the end of the 3rd trimester and staying high until 6–8 weeks postpartum(Hung et al., 2010). Our limited data from non-pregnant controls and normotensive pregnancy are in agreement with this pattern; the reason for its persistence up to 6 weeks after delivery is not clear. The contribution of dietary antioxidants to this overall increase cannot be ruled out. Specific endogenous antioxidants, such as ascorbic acid, vitamin E and zinc, have been analyzed in all three trimesters of normal pregnancies and shown to be inversely related to gestational age, decreasing gradually as pregnancy proceeds toward term (Patil et al., 2007; Anetor et al., 2010). Smoking in mothers has a deleterious effect on both maternal and fetal oxidative and antioxidant status, increasing free radical damage and decreasing antioxidant potential (Chelchowska et al., 2011). Nevertheless, it has been reported that fewer smokers develop PE; those who do, tend to have more severe disease (Broughton Pipkin and Genetics of Preeclampsia Consortium, 2008). We included “smoking” as a factor in our analyses but it did not have an impact on the results in this study.

Our maternal plasma lipid peroxidation data did not show differences between term and preterm deliveries, although there was some increase in the median value in PE women who delivered prematurely (Figure 1); it may be that this parameter is not a sufficiently sensitive measure of severity within the gestational range present in this cohort. Normal pregnancy is not only a state of elevated oxidative stress but also a period of hyperlipidemia, with increases in serum total cholesterol, LDL-cholesterol, HDL-cholesterol, triglyceride, as well as low-density lipoprotein concentrations (Lippi et al., 2007). In PE, these levels are higher than in normal pregnancy (Hubel et al., 1998; Enquobahrie et al., 2004; Zhou et al., 2014) or the same (Hentschke et al., 2013).

Higher concentrations of LDL-cholesterol have been shown by large prospective studies such as the Framingham Heart Study to be positively associated with cardiovascular risk (Gordon et al., 1977), with HDL-cholesterol being protective. In patients with coronary heart disease, LDL-cholesterol is a significant determinant of biomarkers of oxidative stress (i.e., reduced/oxidized glutathione and total antioxidant capacity) (Al-Benna et al., 2006). Therefore, in our study the association between LDL-cholesterol and TBARS 6 weeks postpartum, in hand with the relationship between LDL-cholesterol and the 10 year Framingham CVD risk score, suggests that the damage that ultimately underlies cardiovascular and cerebrovascular insults is already present after pregnancy, albeit, at a low level. The principal role of LDL-cholesterol is the transport of cholesterol to cells, where the delivery is taken up by LDL receptors. The retention of LDL in the arterial intima of the blood vessel initiates an inflammatory response in the vessel wall (Hansson, 2005). In conditions of oxidative stress, the LDL is further modified by oxidation or enzymic attack within the intima and subsequent activation of the lectin-like oxidized LDL receptor-1 (LOX-1) initiates further ROS production, changes in adhesion molecules (which ultimately induces inflammatory cells to adhere to blood vessel walls) and endothelial apoptosis (Chen et al., 2002). OxLDL rises by 260% in early-onset PE (Schreurs et al., 2013); the activation of LOX-1 and subsequent peroxynitrite generation has been postulated as a mechanism for causing blood-brain barrier disruption and increasing risk of life-threatening neurological symptoms. A 2 year follow up study recently published (Zhou et al., 2014), reported that prenatal lipoprotein-associated phospholipase A2 (Lp-PLA2) was elevated in PE, with changes linked to LDL-cholesterol. This enzyme has been associated with coronary endothelial function (Lavi et al., 2007). The incidence of postpartum hypertension in PE was 16% at the 2 year investigation, compared to 1% in the control normotensive pregnancy group; systolic blood pressure positively correlated with lipoprotein-associated phospholipase A_2_ (Lp-PLA2), an enzyme that hydrolyses LDL to phosphatidylcholine and is emerging as a predictive inflammatory marker of cardiovascular risk.

We are continuing to gather postpartum data from our specialist clinic, which will also allow us to analyze EH with superimposed PE. A limitation of this study is the small number of normotensive pregnancy controls, arising because women who enjoyed a normal pregnancy are, understandably, reluctant to attend a specialist clinic. This is being addressed in the larger cohort. Specific markers of oxidative stress would target specific pathways of ROS production and may reveal more regarding mechanisms within each hypertensive disorder, The 8-iso-prostaglandin F_2α_ (PGF_2α_) is known as an *in vivo* biomarker of oxidative stress and can be studied in both plasma and urine to give more complete information; erythrocyte glutathione-S-transferases (Orhan et al., 2003) as well as glutathione peroxidases (Orhan et al., 2003) are also often used as oxidative stress markers. In our observational study we would not be able to demonstrate prevention of oxidative stress upregulation however there are number of published studies that indicate that nutrient supplementation can improve whole blood selenium concentrations (Rayman et al., 2014); protect placental trophoblasts from oxidative stress (Watson et al., 2012; Khera et al., 2013); and has, unproven as yet, potential to decrease the risk of PE (Poston et al., 2011).

Obesity is a risk factor for hypertensive pregnancy and cardiovascular disease. The incidence of obesity and being overweight is a recognized problem in the UK population and the authors are concerned that almost all the women in this study were overweight. This 6 week visit, when the women are coming into contact with the medical profession, is a window of opportunity for counseling as well as research. It would be of great benefit to advise these women on the risks of excess weight and how to lose weight effectively. Specialist clinics with 1 h appointments, like the research clinic described in this study, afford the women time to talk about their previous pregnancy and to discuss lifestyle. In addition, less time-demanding for the women would be telephone-based weight loss intervention which has been shown to be effective and sustainable in women (Kuhlmann et al., 2008; Chasan-Taber et al., 2014; Suksomboon et al., 2014) or by the more-recently explored option of facebook-contact (Kernot et al., 2013) or text-messaging (Steinberg et al., 2013).

There is little doubt now that hypertensive diseases of pregnancy are associated with not only short term complications, but also with longer term cardiovascular and cerebrovascular consequences for both mother and infant. What is not known is whether these women have underlying disease before pregnancy, which is unmasked by the pregnancy, or whether the persistent elevation of oxidative stress and cardiovascular risk factors such as LDL-cholesterol after delivery reflects an impact of pregnancy itself. This study supports the idea that persistent changes are occurring in these conditions and that follow up of these women is vital to provide risk reduction such as dietary advice and antioxidant supplementation.

## Sources of funding

The Nottingham Med-Chi Society generously contributed to the cost of the assay kits.

### Conflict of interest statement

The authors declare that the research was conducted in the absence of any commercial or financial relationships that could be construed as a potential conflict of interest.
